# Demonstration of protein-fragment complementation assay using purified firefly luciferase fragments

**DOI:** 10.1186/1472-6750-13-31

**Published:** 2013-03-28

**Authors:** Yuki Ohmuro-Matsuyama, Chan-I Chung, Hiroshi Ueda

**Affiliations:** 1 Department of Chemistry and Biotechnology, School of Engineering, The University of Tokyo, 7-3-1 Hongo, Bunkyo-ku, Tokyo, 113-8656, Japan; 2 The Japan Society for the Promotion of Science, 8 Ichiban-Cho, Chiyoda-ku, Tokyo, 102-8472, Japan; 3 Chemical Resources Laboratory, Tokyo Institute of Technology, R1-18, 4259 Nagatsuta-cho, Midori-ku, Yokohama, 226-8501, Japan

**Keywords:** Protein-protein interaction, Firefly luciferase, Bioluminescence, Protein fragment complementation assay, Thermostability, In vitro diagnostics

## Abstract

**Background:**

Human interactome is predicted to contain 150,000 to 300,000 protein-protein interactions, (PPIs). Protein-fragment complementation assay (PCA) is one of the most widely used methods to detect PPI, as well as Förster resonance energy transfer (FRET). To date, successful applications of firefly luciferase (Fluc)-based PCA have been reported *in vivo*, in cultured cells and in cell-free lysate, owing to its high sensitivity, high signal-to-background (S/B) ratio, and reversible response. Here we show the assay also works with purified proteins with unexpectedly rapid kinetics.

**Results:**

Split Fluc fragments both fused with a rapamycin-dependently interacting protein pair were made and expressed in *E. coli* system, and purified to homogeneity. When the proteins were used for PCA to detect rapamycin-dependent PPI, they enabled a rapid detection (~1 s) of PPI with high S/B ratio. When Fn7-8 domains (7 nm in length) that was shown to abrogate GFP mutant-based FRET was inserted between split Fluc and FKBP12 as a rigid linker, it still showed some response, suggesting less limitation in interacting partner’s size. Finally, the stability of the probe was investigated. Preincubation of the probes at 37 degreeC up to 1 h showed marked decrease of the luminescent signal to 1.5%, showing the limited stability of this system.

**Conclusion:**

Fluc PCA using purified components will enable a rapid and handy detection of PPIs with high S/B ratio, avoiding the effects of concomitant components. Although the system might not be suitable for large-scale screening due to its limited stability, it can detect an interaction over larger distance than by FRET. This would be the first demonstration of Fluc PCA *in vitro*, which has a distinct advantage over other PPI assays. Our system enables detection of direct PPIs without risk of perturbation by PPI mediators in the complex cellular milieu.

## Background

**Figure 1:**
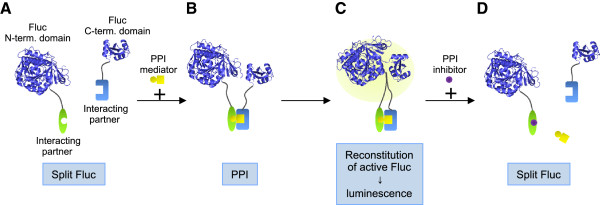
**Principle of Fluc PCA (A) Fluc is dissected into two parts, N-terminal domain and C-terminal domain.** Each fragment is fused to one of interacting partners, respectively. When interacting partners are far apart, luminescence is not observed. (**B**) When PPI occurs, the two Fluc domains come close. (**C**) Upon reconstitution, the luminescence is markedly increased. (**D**) When the PPI inhibitor is added, the luminescence is diminished.

It is estimated that there are about 150,000 to 300,000 protein-protein interactions (PPI) in human interactome [1,2]. In order to discover and investigate PPI, exploration of PPI assays is considered highly important. Among various PPI assays, protein-fragment complementation assay (PCA) is a simple and user-friendly method [3]. In PCA, a reporter protein, such as a green fluorescent protein (GFP) variant [4,5] and a luciferase [6-10] is dissected to a split from, and each split reporter protein is fused to an interacting partner (Figure 1). While the split reporter proteins have low reporter activity when they exist far apart (Figure 1A), they reconstitute and recover the activity when the proteins come close due to the association of interacting partner (Figure 1B). In general, due to its low background activity, PCA displays high signal to background ratio (S/B). Especially, bioluminescence-based PCAs such as split-luciferase reporters shows reversible property [11], in contrast to the irreversible nature of the reassembled GFP-like fluorescent proteins [5]. In addition, since luciferases need no excitation light at shorter wavelength for their detection, the assay enables noninvasive and sensitive optical imaging in living cells and animals.

While PPI is conveniently assayed by PCA *in vivo* and in cultured cells, it is often desirable to be performed also *in vitro*, to know whether the interaction is direct or not, since many PPI mediators, inhibitors and enhancers may exist in the complex cellular milieu. To date, Porter *et al*. successfully utilized a cell-free transcription/translation system for performing firefly luciferase (Fluc)-based PCA *in vitro*[11]. Their probes can be prepared in shorter time period than the probes expressed in *E. coli*, yeast or mammalian cells. However, the assay needs expensive cell-free lysate, and whose components always have a risk to affect PPI. Here we report a PCA using purified interacting proteins fused with split Fluc proteins, to detect PPI in a defined solution. We also examined the stability of the probes, and the detectable distance between the interacting partners. To the best of our knowledge, this will be the first investigation of pure *in vitro* PPI, based on Fluc PCA.

## Results

**Figure 2:**
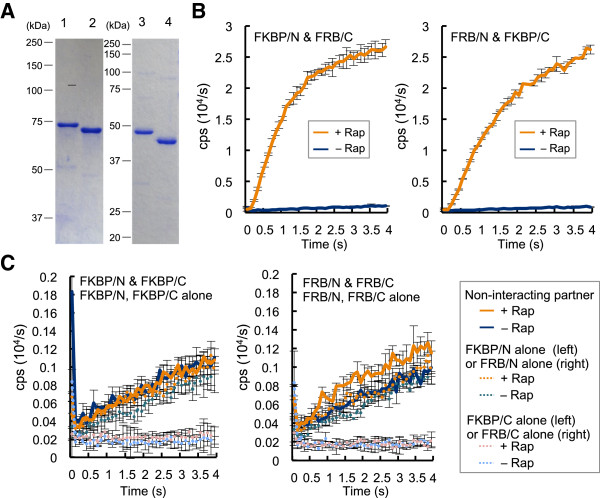
**Fluc PCA using purified probes (A) Purification of the Fluc PCA probes.** Lane 1, FKBP/N; Lane 2, FRB/N; Lane 3, FKBP/C; Lane 4, FRB/C. (**B**) PCA using the purified probes at 50 nM each, with/without equimolar rapamycin (Rap). Average and SD values of three samples are shown. (**C**) Control experiments using non-interacting partners. All the conditions are the same as above.

For the PPI to be investigated, we first selected a well-known interacting domain pair of FKBP12 and FRB. FKBP12 is a 12 kD domain of FK506 binding protein (FKBP), which is able to associate with FKBP-rapamycin-associated protein (FRB) depending on an antibiotic, rapamycin (PPI mediator in Figure 1) [12,13]. To this end, FKBP12 or FRB gene was fused to the 5^′^ of Fluc N-terminal (1–437 in amino acid, aa) or C-terminal domain (394–547 in aa) gene, yielding four types of fusion protein genes (FKBP/N, FKBP/C, FRB/N, and FRB/C). The genes were inserted to pET32b vector, and the thioredoxin-fused proteins were expressed in the soluble fraction of *E. coli* BL21(DE3, pLysS) and purified by an immobilized metal affinity chromatography (Figure 2A). The two interacting pairs (FKBP/N - FRB/C and FKBP/C - FRB/N) were mixed at 50 nM each, and equimolar rapamycin was added to the mixtures. Just after adding the two substrates ATP and luciferin, the luminescent intensity was measured by a luminometer at 0.1 s intervals for 4 s. As a result, the intensity of the interacting pairs added with rapamycin showed a marked increase. In contrast, the pairs without rapamycin displayed very low luminescence (Figure 2B). In addition, non-interacting protein pairs (FKBP/N - FKBP/C, and FRB/N - FRB/C), as well as each fusion protein alone exhibit very low luminescence even in the presence of rapamycin (Figure 2C). The results clearly showed that the PPI can be specifically detected with high S/B ratio using purified probes.

**Figure 3:**
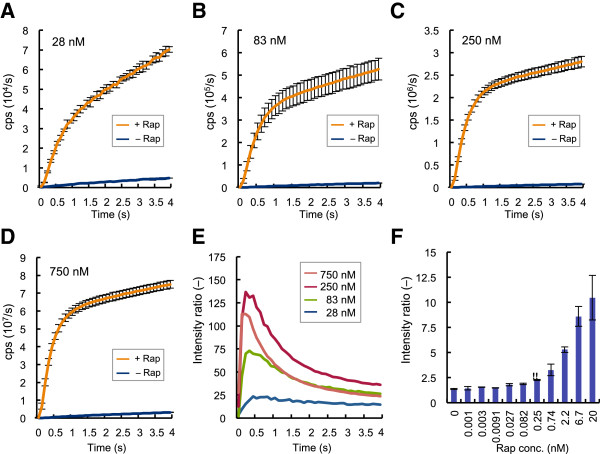
**Concentration dependency of Fluc PCA and sensitivity to rapamycin (A-D) Fluc PCA with FRB/N and FKBP/C with/without equimolar rapamycin at indicated concentrations.** Average and SD of three samples are shown. (**E**) Concentration dependency of signal/background ratio. (**F**) Rapamycin dose–response using 50 nM each probes. ** : Limit of detection showing higher signal-1SD than the background signal +2SD.

To assess the degree of spontaneous Fluc reconstitution, the assay with one of the best interacting pairs FRB/N and FKBP/C was performed at several probe concentrations (Figure 3). At all the concentrations tested, the luminescent signals increased rapamycin-dependently (Figure 3, A-D). The resulting maximum signal/background (S/B) ratio increased to as high as 130 when at the probe concentration increased up to 250 nM, while the ratio at 750 nM was slightly lower (Figure 3E). This was possibly because the high probe concentration rather promoted spontaneous Fluc reconstitution without rapamycin. When the rapamycin dose–response at 50 nM probe concentration was examined, the limit of detection was determined as 250 pM (Figure 3F).

For Fluc PCA, several successful split sites are reported to date. To investigate on this issue, another pair of N-terminal domain (1–398) together with the same C-terminal domain (394–547) was employed according to a previous attempt that gave good response *in vivo* (Additional file 1: Figure S1) [14]. We could express and purify the new N-domain similarly (Additional file 1: Figure S1A), and obtained clear rapamycin-dependent signals at several concentrations of FRB/N(1–398) – FKBP/C pair (Additional file 1: Figure S1, B-E). However, compared with the FRB/N(1–437) – FKBP/C(394–547) pair, the signal intensity was lower, and the resultant S/B ratio was rather unstable, albeit not low (Additional file 1: Figure S1F). This was probably because of the instability of the N-domain (1–398), which is reflected to its degradation products observed in Additional file 1: Figure S1A.

**Figure 4:**
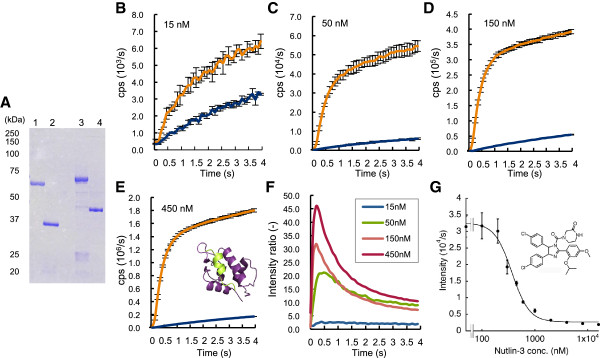
**Detection of p53 - Mdm2 interaction (A) SDS-PAGE of p53 and Mdm2 fusion probes.** Lane 1, p53/N; Lane 2, p53/C; Lane 3, Mdm2/N; Lane 4, Mdm2/C. (**B-E**) Fluc PCA using an interacting partner p53/C and Mdm2/N (orange) or a non-interacting partner p53/C and p53/N (dark cyan) each at indicated concentrations. Average and SD of three samples are shown. (**F**) Signal/background ratios of Fluc PCA using p53/C and Mdm2/N. (**G**) Effect of Nutlin-3 on p53-Mdm2 interaction. Average and SD of three samples are shown.

Since rapamycin-dependent FKBP-FRB interaction is one of very strong protein-protein interactions [15], another cellular interaction of Mdm2 and p53 was investigated (Figure 4). Mdm2 oncoprotein is a cellular inhibitor of the p53 tumor suppressor in that it can bind the trans-activation domain of p53 and down-regulates its ability to activate transcription. In certain cancers, mdm2 amplification is a common event and contributes to the inactivation of p53 [16]. The probes fused to the trans-activation domain of p53 and Mdm2 (p53/C, p53/N, Mdm2/C and Mdm2/N) were constructed in the same way as FKBP/C and FRB/N (1–437) (Figure 4A). This time, both the signal intensity and S/B ratio elevated depending on the concentration of the probes (Figure 4, B-F). Then we investigated whether the *in vitro* Fluc PCA could detect the reversibility of interaction. After we mixed the probes p53/C and Mdm2/N at 100 nM each, Nutlin-3, a known inhibitor of p53-Mdm2 interaction, was added and measured for the Fluc activity (Figure 4G). The result clearly showed a Nutlin-3 concentration dependent luminescence inhibition with calculated IC_50_ of 368 ± 25 nM. Although the value is higher than the reported one (90 nM) [17], this might be due to the racemization of Nutlin-3 used. Taken together, these results clearly showed the generality of the assay to detect PPIs including transient interactions.

Due to the nature of split enzyme, we reasoned that the instability of the purified probes could be a possible demerit of Fluc-based PCA. To examine the stability of the probes, the mixtures of 100 nM FKBP/C and 100 nM FRB/N with or without equimolar rapamycin were incubated at 37°C for 0–60 min, and applied to the luminescence assay (Additional file 2: Figure S2). As a result, the incubation resulted in a time-dependent decrease in luminescent intensity of the pair added with rapamycin (Additional file 2: Figure S2A). As short as 15 min incubation resulted in the activity loss to less than one-third of that before incubation. The longer incubation for 60 min resulted in virtually no signal difference between the pairs with and without rapamycin (Additional file 2: Figure S2, B and C). These results clearly indicate that the Fluc PCA probes have a limited stability in this purified milieu. However, the results might also reflect their limited stability *in vivo* or in lysate, which is probably sequestered by the newly synthesized probes.

**Figure 5:**
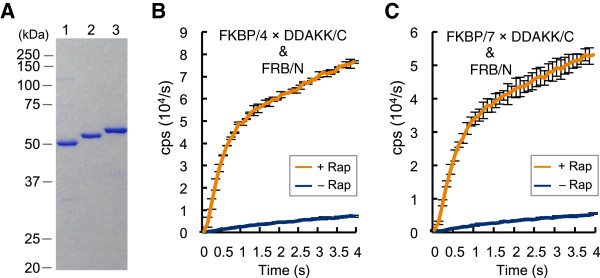
**Insertion of helix linkers (A) SDS-PAGE of the probes inserted with helix linkers.** Lane 1, FKBP/C; Lane 2, FKBP / 4 × DDAKK /C; Lane 3, FKBP/ 7 × DDAKK /C. (**B**) PCA using the probe with 4 × DDAKK. The probes (100 nM each) with/without equimolar rapamycin was used (n = 3). (**C**) PCA using the probe with 7 × DDAKK. The condition is the same as in (**B**).

Förster resonance energy transfer (FRET) is an excellent PPI detection method both *in vitro* and *in vivo*, and regarded as a standard tool in this area [18]. However, FRET has a fundamental limitation that it only occurs over rather short distance of several nm between the donor and the acceptor fluorophores, depending on Förster distance *R*_0_, which is determined *a priori* by the dye combination used [19,20]. Therefore, the interaction between proteins with large dimensions is a challenging task for FRET, due to the possible long distance between the attached dyes including fluorescent proteins. To compare Fluc PCA with FRET, we examined the effect of distance between interacting partners. Previously, we introduced several helix-forming peptide linkers with various lengths between EGFP and EBFP, and those linkers showed the ability to control the distance between interacting partners [21]. According to this, a similar helix-forming linker, 4-repeats of Asp-Asp-Ala-Lys-Lys (4 × DDAKK), was inserted between FKBP12 and Fluc C-terminal domain. The SDS polyacrylamide gel electrophoresis of the fusion proteins (Figure 5A) suggests effective separation of FKBP12 and Fluc C-terminal domain by the inserted linkers. Using 100 nM each mixture of FKBP/4 × DDAKK/C, FRB/N and rapamycin, PPI was detected with a high S/B ratio with a slight reduction compared with the pairs without helical linkers (Figure 5B). In addition, when a linker with 7 repeats of Asp-Asp-Ala-Lys-Lys (7 × DDAKK) was inserted between FKBP12 and Fluc C-terminal domain, high S/B ratio was still maintained (Figure 5, A and C).

**Figure 6:**
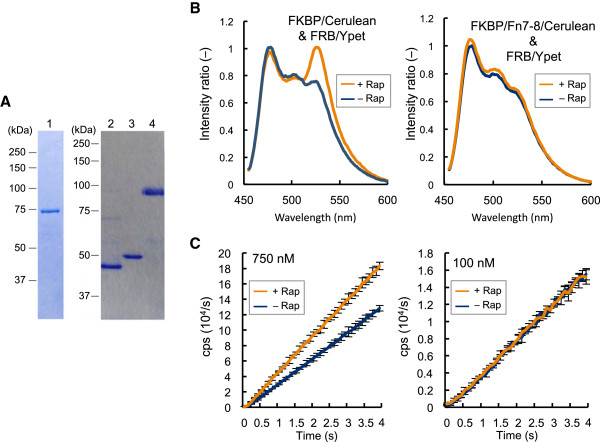
**Comparison with FRET in the presence of a rigid linker (A) SDS-PAGE of the PCA/FRET probes with or without Fn7-8 domains.** Lane 1, FKBP/Fn7-8/C; Lane 2, FRB/Ypet; Lane 3, FKBP/Cerulean; Lane 4 FKBP/Fn7-8/Cerulean. (**B**) FRET assays. Left: the assay using the probe pair without insertion. Right: the assay using the probe pair inserted with Fn7-8. (**C**) Fluc PCA using the probe inserted with Fn7-8. The probe/rapamycin concentrations of 750 nM (left) and 100 nM (right) were investigated (n = 3).

For a further examination, the 7th and 8th domains of fibronectin type III (Fn7-8) with a 7 nm N-C terminal distance was inserted between FKBP12 and Fluc C-terminal domain, as a rigid linker. From the SDS-PAGE of purified proteins, while the mobility of the reporter was lowered by the insertion of Fn7-8, no significant reduction in the yield and purity was observed (Figure 6A, Lanes 1 and 4). Ohashi et al. reported that the insertion of Fn7-8 domains between the fluorescent protein pair CyPet and Ypet results in barely detectable FRET signal. [22]. To confirm this, Fn7-8 was inserted between FKBP12 and a brighter CFP variant Cerulean (FKBP/Fn7-8/Cerulean) (Figure 6A, Lanes 2 and 3). As expected, very weak FRET signal derived of FKBP/Fn7-8/Cerulean and FRB-fused Ypet (FRB/Ypet) was observed, although a distinct FRET-derived peak was observed for the construct without Fn7-8 insertion (Figure 6B).

On the other hand, the PCA pair of FKBP/Fn7-8/C and FRB/N at 750 nM displayed a moderate but significant S/B ratio, clearly showing its superiority over FRET, although the observed rapamycin dependency was not so clear when reduced amounts (100 nM) of the probes were used (Figure 6C). Nevertheless, the Fluc PCA system was shown to work for detecting PPI over longer distance, which was not detectable by FRET.

## Discussion

Previously, Fluc-based PCA has been utilized as a sensitive tool to investigate PPI *in vivo* and in cultured cells. However, considering possible disturbance by the co-existing components acting as PPI mediators, enhancers and inhibitors, PPI assay *in vitro* will also give us invaluable information. Furthermore, the *in vitro* assay has the possibility to study PPIs between pathogen-derived cytotoxic proteins and host proteins, which are normally impossible to perform *in vivo*. In this study, we showed that Fluc PCA also works with purified elements. The system displayed high S/B ratio and unexpectedly rapid response (~1 s). In PCA, the background signal due to nonspecific activation of the reporter can be a problem. As in Figure 2C, luminescent intensities of non-interacting pairs were slightly higher than those of single probe alone, probably reflecting the nonspecific reconstitution of split Fluc. However, when the specific PPI was induced by rapamycin, it resulted in markedly higher signal, giving high S/B ratio. Therefore, in practice the nonspecific signals hardly interfere with the PPI detection (Figure 2).

The only but not easily avoidable drawback of this system is the instability of probe as shown in Additional file 2: Figure S2. Apparently, the system is not very suitable for large-scale screening that needs longer sample preparation time. However, proper cooling of the reagents and samples during the preparation period will surely reduce such inactivation. Therefore, we think that the system has a potential for low-cost and high-throughput drug screenings. On the other hand, a distinct merit of this system is robustness to the interaction detection over longer distance. In fact, insertion of either long helical linker or a rigid large protein domain did not deteriorate PPI detection, while FRET signal was barely detectable when Fn7-8 was inserted (Figure 6B). These results show a lesser limitation in distance between interacting partners in PCA than in FRET. Therefore, this system will be available for the interactions between large proteins that cannot be assayed by FRET. While the reason for observed nonspecific PPI signal remains unclear, it may be due to the interaction between the inserted sequence and the other probe. In addition, we could observe the higher S/B ratio with the higher concentration of a probe set (Figure 6C). Use of higher probe concentration is desirable to assay larger interacting partners. The nearly straight shape of the kinetic curve observed might reflect the large dimension of Fn7-8, which would lower the reconstitution efficiency and subsequent luminescent reaction rate.

Collectively, considering the demerits and the merits, Fluc PCA using purified elements enables us a rapid and convenient detection of direct PPI with high confidence.

## Conclusions

The feasibility of Fluc PCA *in vitro* using purified elements was demonstrated for the first time. The assay was found to have high S/B ratio, and allow the detection of larger size of interacting partner than detectable by FRET. In spite of relatively weak thermostability, the assay will be applicable to handy detection of various PPIs *in vitro* including *in vitro* diagnostics and drug screening.

## Methods

### Materials

ATP and D-luciferin (LH_2_) were from Sigma, St. Louis, MO. MOPS (3-(N-morpholino)propanesulfonic acid) was from Dojindo, Kumamoto, Japan. Rapamycin was from Wako Pure Chemical Industries, Osaka, Japan or LKT Laboratories, St. Paul, MN. Synthetic genes for *E. coli* codon-optimized human FKBP12, appended with *Nco* I / *Sfi* I and *Not*I sites at the 5’ and 3^′^ ends, respectively, were from Mr Gene GmbH, Regensburg, Germany. The plasmid pFH154 encoding human fibronectin cDNA was from Health Science Research Resources Bank (HSRRB), Osaka, Japan. Other reagents in the highest grade available were from Wako Pure Chemical Industries unless otherwise indicated.

### Construction of FKBP/FRB fused Fluc fragments

The DNA fragment encoding Fluc was obtained by PCR using pGEX-Ppy vector [23] as a template, and primers LucNotG4SB (5^′^- gg cgc gcc GCG GCC GCC GGT GGT GGT GGT AGC ATG GAA GAC GCC AAA AAC ATA AAG-3^′^) encoding a G_4_S linker and *Not*I site (underlined) and LucXhoF (5^′^- g gcg cgc CTC GAG CTT TCC GCC CTT CTT GGC CT- 3^′^) containing *Xho*I site (underlined). Similarly, N- and C-terminal domain genes were amplified with primers LucNotG4SB and Luc437XhoF (5^′^-g gcg cgc CTC GAG GCG GTC AAC TAT GAA GAA GTG- 3^′^), and Luc394NotG4SB (5^′^- gg cgc gcc GCG GCC GCC GGT GGT GGT GGT AGC GGA CCT ATG ATT ATG TCC GG-3^′^) and LucXhoF, respectively. The amplified fragments were cloned into pET32b (Merck, Darmstadt, Germany) between the *Not*I and *Xho*I sites, to give pET32/Fluc, pET32/FlucN and pET32/FlucC, respectively. The synthetic genes encoding FKBP12 and FRB cDNAs were digested with *Nco*I and *Not*I*,* and the digested fragments inserted in pET32/FlucN and pET32/FlucC digested with the same enzymes each other, to give pET32/FKBP/FlucN, pET32/FKBP/FlucC, pET32/FRB/FlucN and pET32/FRB/FlucC.

### Construction of FRB fused FlucN(1–398) fragments

The DNA fragment encoding Fluc N-terminal domain (1–398) was obtained by PCR using pET32/FRB/FlucN as a template, and primers LucNotG4SB and Luc398XhoF (5^′^- t gtt tac ata CTC GAG cat aat cat agg tcc tct tac- 3^′^) containing *Xho*I site (underlined). The amplified fragments were cloned into pET32/FRB/FlucN between the *Not*I and *Xho*I sites, to give pET32/FRB/FlucN (1–398).

### Construction of p53/Mdm2 fused Fluc fragments

The DNA fragment encoding transactivation domain of p53 (residue 15–29) and two restriction sites (underlined) was obtained by thermal cycling using following oligonucleotides: p53NcoBack (5^′^-gg aat tCC ATG GCT AGT CAG GAA ACA TTT TCA GAC CTA TGG AAA C-3^′^) and p53NotFor (5^′^- g gga ttc tGC GGC CGC GTT TTC AGG AAG TAG TTT CCA TAG GTC TG-3^′^). Mdm2 gene (residue 17–125) was obtained by PCR with human Mdm2 gene as a template and mdm2NocBack (5^′^-gg aat tCC ATG GCT TCG GAA CAA GAG ACC C-3^′^) and mdm2NotFor (5^′^-g gaa ttc tGC GGC CGC CTG CTG ATT GAC TAC TAC C-3^′^) as primers. The amplified fragments were digested with *Nco*I and *Not*I, and inserted to pET32/FKBP/FlucC and pET32/FRB/FlucN digested with the same enzymes, to give pET32/p53/FlucN, pET32/p53/FlucC, pET32/Mdm2/FlucN and pET32/Mdm2/FlucC.

### Insertion of 4 × DDAKK between FKBP12 and FlucC

The two oligonucleotides DDAKK4_NotBack2 (5^′^ –g gaa ttc GCG GCC GCA GAT GAT GCT AAA AAA GAT GCT AAA AAA GAT GAT GCC AAG AAG GAC GAC GC- 3^′^) containing *Not*I site (underlined), and DDAKK4_EagFor2 (5^′^ –g gaa ttC GGC CGA TTT TTT AGC ATC ATC TTT TTT CGC GTC GTC CTT CTT GGC- 3^′^) containing *Eag*I site (underlined) were annealed and extended using a thermal cycler. The fragment was digested with *Eag*I, and inserted into pET32/FKBP/FlucC digested with *Not*I, to give pET32/FKBP/4 × DDAKK/FlucC.

### Insertion of 7×DDAKK between FKBP12 and FlucC

pET32/FKBP/4 × DDAKK/FlucC was amplified with the primers, DDAKK_VectorNotB (5^′^-GA CGA CGC CAA AAA AGA TGA TGC CAA GAA GG-3^′^), and DDAKK_VectorNotF (5^′^-CT TTT TTA GCA TCA TCT GCG G-3^′^). The 4 × DDAKK fragment inserted in pET32/FKBP/4 × DDAKK/FlucC was amplified with the primers DDAKK_LinkerB (5^′^ –GA TGA TGC TAA AAA AGA TG- 3^′^) and DDAKK_LinkerF (5^′^-TT TTT TGG CGT CGT CTT TTT TCG CGT CGT C- 3^′^). The two amplified fragments were connected using In-Fusion HD cloning kit (Takara-Bio, Shiga, Japan), to obtain pET32/FKBP/8 × DDAKK/FlucC, which resulted in unexpected acquisition of pET32/FKBP/7 × DDAKK/FlucC.

### Construction of FKBP/FRB fused with fluorescent proteins

The cDNAs for Ypet on pYpet-His (kindly provided by Dr. PS Daugherty) [24] and Cerulean [25] made from pEBFP-N1 plasmid (Clontech, Takara-Bio) were amplified using specific primers with 5′-terminal *Not*I and 3^′^-terminal *Xho*I sites, digested with *Not*I and *Xho*I, and inserted into pET32/FKBP/FlucN and pET32/FRB/FlucN digested with the same enzymes, to give pET32/FKBP/Cerulean and pET32/FRB/Ypet.

### Insertion of Fn7-8 as a rigid linker

The sequence for Fn7-8 was amplified from human fibronectin cDNA with the primers Fn7EagBack (5^′^-g gaa ttC GGC CGC ACC ATT GTC TCC ACC AAC AAA C- 3^′^) and Fn8EagFor (5^′^-g gaa ttC GGC CGA TGT TTT CTG TCT TCC TCT AAG- 3^′^) each containing an *Eag*I site. The amplified fragment was digested with *Eag*I, and was inserted into pET32/FKBP/FlucC and pET32/FKBP/Cerulean digested with same enzyme, respectively.

### Expression and purification of probe proteins

All the fusion proteins were expressed in *E. coli* BL21 (pLysS, DE3) (Novagen) as a thioredoxin and hexahistidine-tagged protein. To purify the expressed fusion proteins, Talon metal affinity resin (Clontech) was used according to the manufacturer’s instruction. Concentration of the purified protein was determined by CBB-stained SDS-PAGE co-loaded with various concentrations of BSA as a concentration standard. The protein added with final 15 % glycerol was stored at −80°C before use.

### Detection of PCA

The purified probe proteins with or without rapamycin were suspended in 100 mM MOPS, 10 mM MgSO_4_, pH 7.3. The mixture (50 μl each) was dispensed to a well of 96-well half well white plate (Corning-Costar, NY, USA). The light intensity was measured immediately after injection of 50 μl 2 × substrate solution (40 mM ATP and 150 μM LH_2_ in 100 mM MOPS, 10 mM MgSO_4_, pH 7.3) with a periodical integration for 0.1 s using a luminometer Phelios AB-2350 (ATTO, Tokyo, Japan).

### FRET assay

Fluorescence spectra were measured by F-2500 fluorescence spectrophotometer (Hitachi High-Technologies, Tokyo, Japan). Samples were diluted in 250 μl PBS, pH 7.4. The mixture of FKBP/cerulean or FKBP/Fn/cerulean and FRB/Ypet (40 nM each) was excited at 433 nm, and the fluorescent spectra at 455–600 nm were recorded in the presence and absence of 40 nM rapamycin.

## Abbreviations

FKBP: FK506 binding protein; Fluc: firefly luciferase; Fn7-8: 7–8 domains of fibronectin type III; FRB: FKBP-rapamycin-associated protein; FRET: fluorescence resonance energy transfer; GFP: green fluorescent protein; MOPS: 3-(N-morpholino)propanesulfonic acid; PCA: protein-fragment complementation assay; PPI: protein-protein interaction.

## Competing interests

The authors declare that they have no competing interests.

## Authors’ contributions

YOM and HU designed and performed the research and wrote the manuscript. CIC performed the research and helped writing the manuscript. All authors read and approved the final manuscript.

## Supplementary Material

Additional file 1: Figure S1 Fluc PCA using a probe split at different site FRB/N (1-398). (A) SDS-PAGE of purified FRB/N (1-398). (B-E) Fluc PCA using FRB/N (1-398) and FKBP/C in 15, 50, 150, and 450 nM. (F) Signal/background ratios of Fluc PCA using FRB/N (1-398) and FKBP/C. Click here for file

Additional file 2: Figure S2 Examination of the probe stability. After incubating 100 nM each of the probes with equimolar rapamycin (A) or without rapamycin (B) for 0 (lemon), 15 (yellow), 30 (orange) and 60 min (red) at 37°C, PCA was performed as before. In (B), smaller scale of the vertical axis is used in the inset. (C) Comparison of the luminescent intensities with/without rapamycin after 60 min incubation. Click here for file
