# Supplementary material for: Demonstration of protein-fragment complementation assay using purified firefly luciferase fragments
Source: BMC Biotechnol. 2013 Mar 28;13:31. doi: 10.1186/1472-6750-13-31 (PMC3626928; doi:10.1186/1472-6750-13-31)
Supplement: Additional file 1: Figure S1 — Fluc PCA using a probe split at different site FRB/N (1-398). (A) SDS-PAGE of purified FRB/N (1-398). (B-E) Fluc PCA using FRB/N (1-398) and FKBP/C in 15, 50, 150, and 450 nM. (F) Signal/background ratios of Fluc PCA using FRB/N (1-398) and FKBP/C. [file 1472-6750-13-31-S1.pdf]

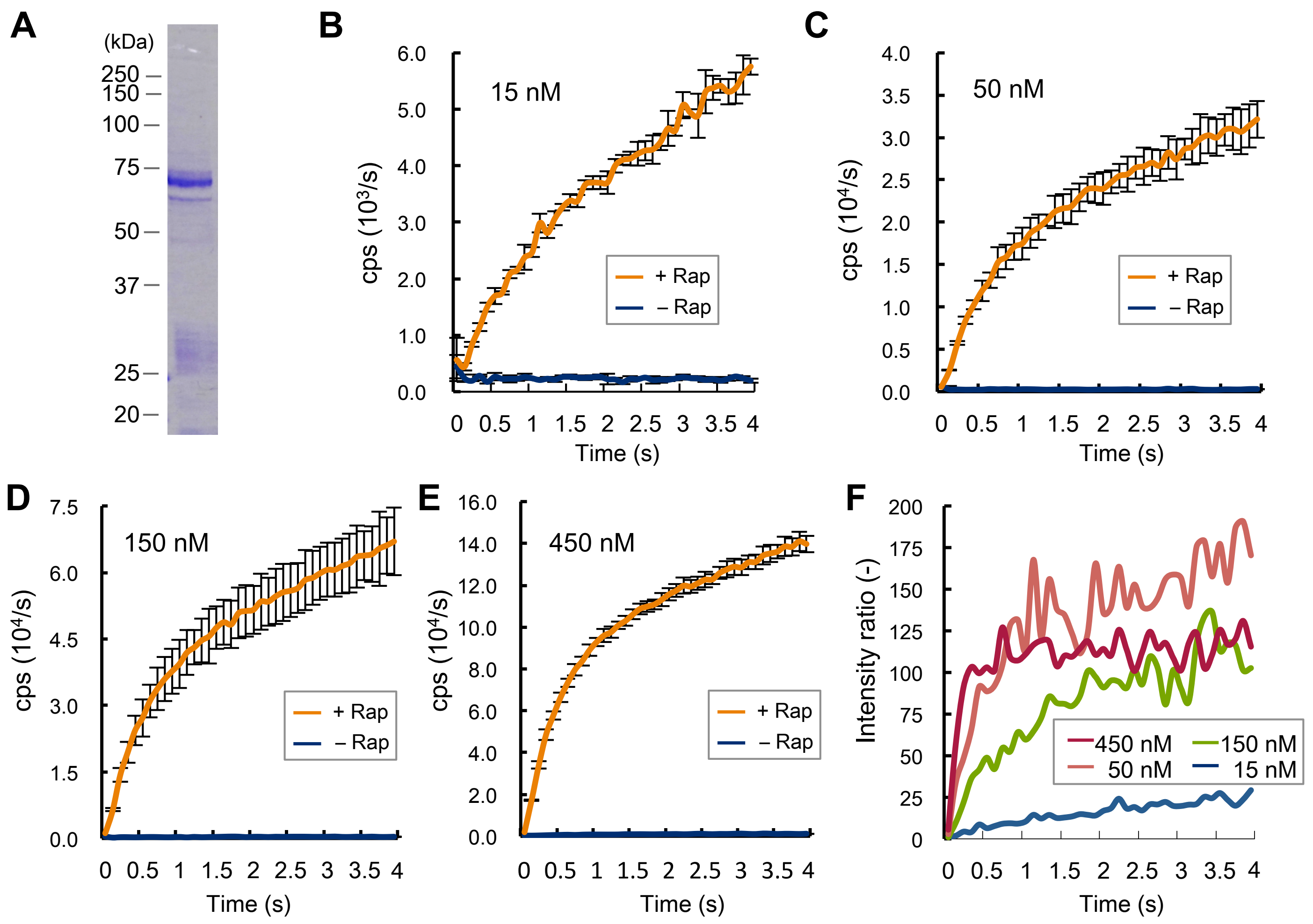


**Supplementary Figure S1 - Fluc PCA using a probe split at different site FRB/N (1-398)**
(A) SDS-PAGE of purified FRB/N (1-398). (B-E) Fluc PCA using FRB/N (1-398) and FKBP/C in 15, 50, 150, and 450 nM. (F) Signal/background ratios of Fluc PCA using FRB/N (1-398) and FKBP/C.
