# Supplementary material for: Demonstration of protein-fragment complementation assay using purified firefly luciferase fragments
Source: BMC Biotechnol. 2013 Mar 28;13:31. doi: 10.1186/1472-6750-13-31 (PMC3626928; doi:10.1186/1472-6750-13-31)
Supplement: Additional file 2: Figure S2 — Examination of the probe stability. After incubating 100 nM each of the probes with equimolar rapamycin (A) or without rapamycin (B) for 0 (lemon), 15 (yellow), 30 (orange) and 60 min (red) at 37°C, PCA was performed as before. In (B), smaller scale of the vertical axis is used in the inset. (C) Comparison of the luminescent intensities with/without rapamycin after 60 min incubation. [file 1472-6750-13-31-S2.pdf]

Fig. S2

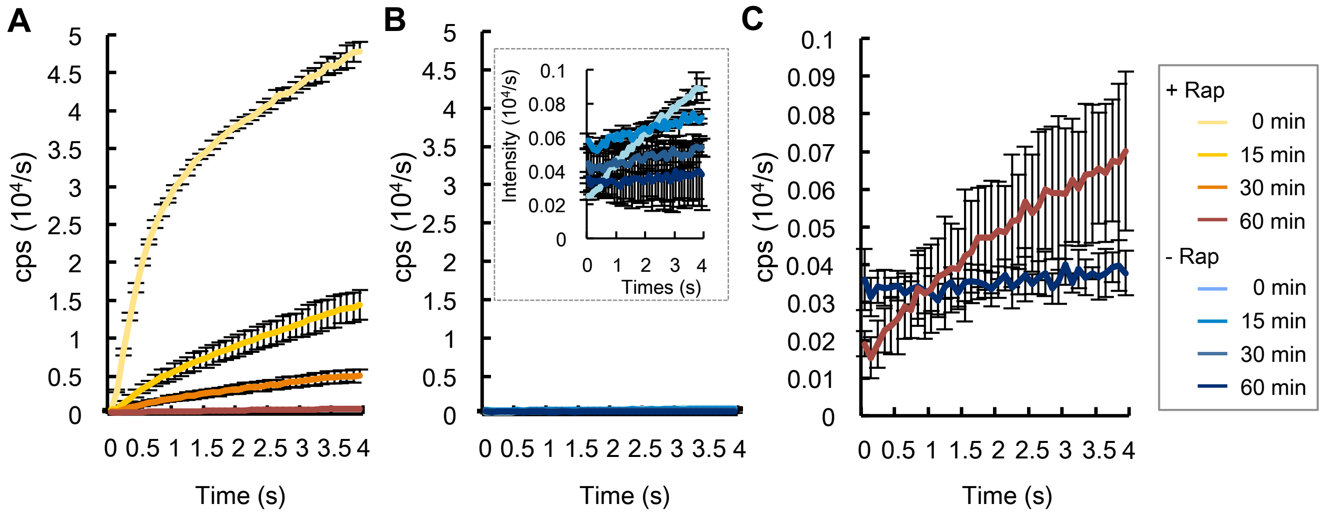

Supplementary Figure S2 - Examination of the probe stability

After incubating 100 nM each of the probes with equimolar rapamycin (A) or without rapamycin (B) for 0 (lemon), 15 (yellow), 30 (orange) and 60 min (red) at 37°C, PCA was performed as before. In (B), smaller scale of the vertical axis is used in the inset. (C) Comparison of the luminescent intensities with/without rapamycin after 60 min incubation.
